# Cancer Care to Ukrainian War Refugees in Poland

**DOI:** 10.1001/jamanetworkopen.2023.21967

**Published:** 2023-07-06

**Authors:** Stanislaw Klek, Katarzyna Chrobak-Kasprzyk, Klaudia Machnicka, Kamila Kret, Aleksandra Litewka, Nicole Kantor, Janusz Rys

**Affiliations:** 1 Surgical Oncology Clinic, Maria Sklodowska-Curie National Research Institute of Oncology, Krakow Branch, Garncarska, Krakow, Poland

## Abstract

This cohort study evaluates cancer care provided to Ukrainian war refugees in Poland.

## Introduction

The Russian invasion disrupted all Ukrainian medical services; hence, effective cancer care became difficult, and the cancer burden was high.^1,2^ With more than 7.5 million Ukrainian residents coming to Poland as of November 1, 2022, and 1.3 million staying permanently, local cancer care started to be tested. Within the first 6 weeks, the National Cancer Institute in Krakow (NIO) was approached by 112 patients.^3,4^ The center adapted swiftly to the new situation by installing hotlines, hiring interpreters, and transforming registration offices and outpatient units. The aim of this study was to analyze whether the Polish medical system was capable of providing appropriate care to refugees without limiting care for local patients.

## Methods

This cohort study was a retrospective analysis of 912 medical records, including 304 Ukrainian refugees who were consulted and/or treated at NIO during the first 6 months after the beginning of the war in Ukraine (from February 24 to August 24, 2022). The National Cancer Research Institute Ethics Committee approved the study. This report follows the Strengthening the Reporting of Observational Studies in Epidemiology (STROBE) reporting guideline for cohort studies. All available medical documents, in any available language, were analyzed. Patients agreed to participate in the study upon admission to NIO by providing written consent. Descriptive statistics were used to analyze the data. One-sided χ^2^ tests of independence were completed, and differences at *P* < .05 were regarded as statistically significant.

## Results

A total of 304 patients (240 female [78.9%]; mean [IQR] age 55 [18-85] years) accounted for 30% of all new patients referred to the Institute and increased the overall number of patients at the NIO by 9.7%. The mean (SD) time from the first visit to the initiation of therapy was 27.7 (7.8) days for the refugees and 26.1 (10.2) days for Polish citizens. Patients with breast cancer constituted the largest group (90 patients [31.5%]), followed by patients with colon cancer (15 patients [5.5%]), lung cancer (14 patients [4.9%]), cervix cancer (14 patients [4.9%]), and melanoma (14 patients [4.9%]) (Figure).

**Figure.**
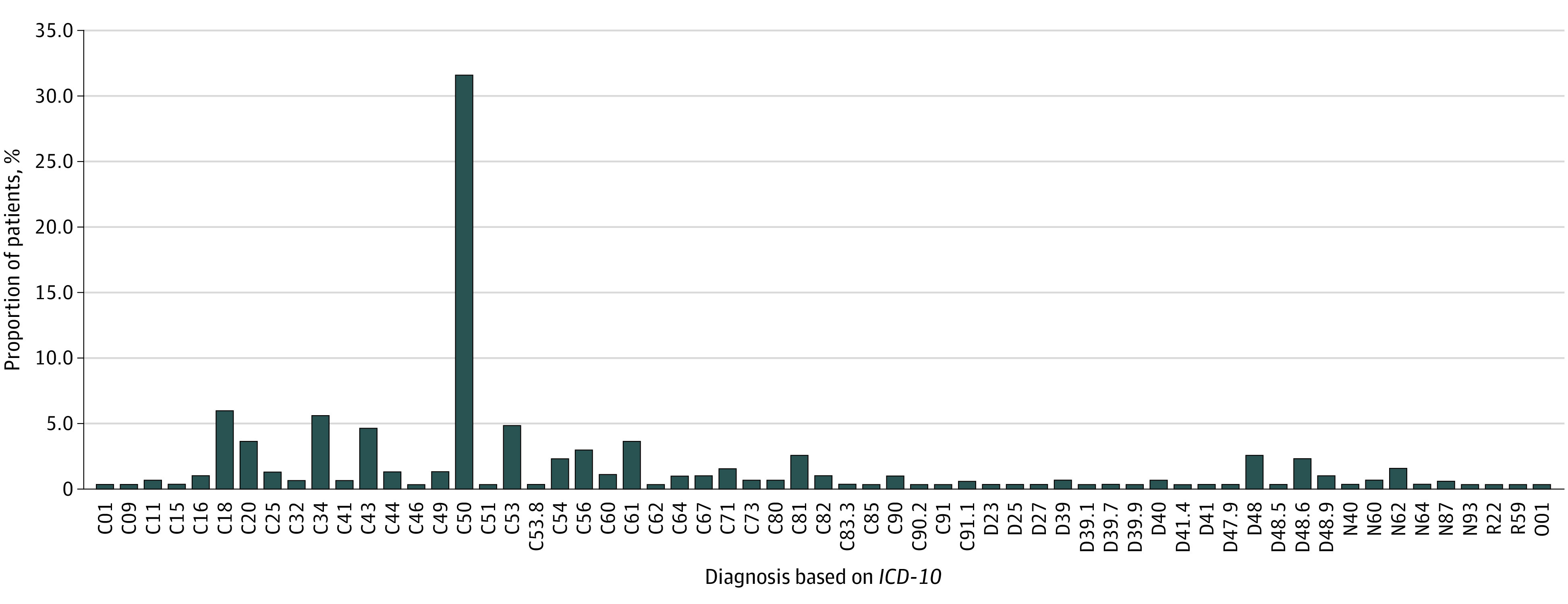
Diagnoses (*International Statistical Classification of Diseases and Related Health Problems, Tenth Revision [ICD-10]*)

Of 304 cases, 208 refugees (68.4%) continued therapy initiated in Ukraine. In 231 cases (76%), medical records were available; however, a reliable pathology report was not available in 15 cases (4.9%). In most cases (160 patients [52.5%]) there was a need for translation.

A total of 1750 consultations were performed within 6 months. Treatment options for nonsurgical oncology included chemotherapy (76 cases [36.9%]), radiotherapy (57 cases [27.7%]), hormonotherapy (44 cases [21.4%]), and immunotherapy (27 cases [13.1%]) (Table). Some patients (41 patients [13.5%]) lived in Ukraine during their therapy. One hundred thirty seven patients (45.1%) stayed in Poland after treatment. Breast conserving surgery was the most frequent surgical procedure (20 patients [38.2%]), followed by mastectomy (12 patients [20%]) (Table).

**Table.** Total Number of First-Time Patients and Types of the Most Frequent Surgical Procedures

Department and Procedure	Refugees, No. (%)
Department	
Surgical oncology	98 (7.7)
Clinical oncology	104 (18.9)
Urology	18 (3.8)
Gynecology	55 (14.6)
Hematology	15 (10.8)
Radiotherapy	14 (4.6)
Total	304 (9.7)
Procedure	
Quadrantectomy plus sentinel lymph node biopsy	14 (27.3)
Quadrantectomy plus ALND	6 (10.9)
Mastectomy plus ALND (Madden procedure)	6 (10.9)
Transurethral resection of bladder tumor	6 (10.9)
Simple mastectomy plus SNB	5 (9.1)
Hysterectomy	5 (9.1)
Nipple sparing mastectomy with simultaneous reconstruction	3 (7.2)
Melanoma: excision of the scar plus SNB	3 (7.3)
Hemicolectomy	3 (5.5)
Anterior rectal resection	3 (5.5)
Gastrectomy	1 (1.8)

Abbreviations: ALND, axillary lymph node dissection; SNB, sentinal node biopsy.

### Discussion

Authors are aware of potential limitations of the study, such as selection bias; the analyzed population was composed of people capable of crossing the border and healthy enough to arrive. Nonetheless, the study suggests that even with a substantial number of new cases, effective cancer care could be delivered provided that structural changes were applied. Cancer care of local citizens was not adversely affected, even with a high volume of patients.

## Notes

Supplement.
Data Sharing Statement
