# Supplementary material for: Cancer Care to Ukrainian War Refugees in Poland
Source: JAMA Netw Open. 2023 Jul 6;6(7):e2321967. doi: 10.1001/jamanetworkopen.2023.21967 (PMC10326634; doi:10.1001/jamanetworkopen.2023.21967)
Supplement: Supplement. — Data Sharing Statement [file jamanetwopen-e2321967-s001.pdf]

Data Sharing Statement

Klek. Cancer Care to Ukrainian War Refugees in Poland. *JAMA Netw Open*. Published July 06, 2023. doi:10.1001/jamanetworkopen.2023.21967

Data
Data available: Yes
Data types: Deidentified participant data
How to access data: pesonal contact
When available: With publication

Supporting Documents
Document types: Statistical/analytic code
How to access documents: email: st.klek@gmail.com
When available: With publication

Additional Information
Who can access the data: anyone requesting the data
Types of analyses: for a specified purpose
Mechanisms of data availability: after approval of a proposal
Any additional restrictions: none
